# Psychosocial Burden in Parents of Pediatric Liver Transplant Recipients

**DOI:** 10.3390/healthcare14101384

**Published:** 2026-05-19

**Authors:** Serkan Suren, Deniz Yavuz Baskiran, Irem Tulum, Adil Baskiran, Sezai Yilmaz

**Affiliations:** 1 Child and Adolescent Psychiatry Practice, Samsun 55200, Türkiye; drserkansuren@hotmail.com (S.S.); iremttulum@gmail.com (I.T.); 2 Liver Transplantation Institute, Inonu University, Malatya 44210, Türkiye; dr.adil.baskiran@gmail.com (A.B.); sezai.yilmaz@inonu.edu.tr (S.Y.)

**Keywords:** liver transplantation, parental stress, anxiety, depression, sleep quality

## Abstract

**Highlights:**

**What are the main findings?**

- Pediatric liver transplantation was associated with a considerable psychosocial burden in parents, reflected by stress, sleep problems, depression, and anxiety.
- Perceived stress emerged as an independent predictor of depressive and anxiety symptoms, while longer time since transplantation was associated with lower psychological distress.

**What are the implications of the main findings?**

- Psychosocial screening and mental health support should be considered as part of routine follow-up for parents of pediatric liver transplant recipients.
- Early identification and management of parental stress may improve family adaptation and overall post-transplant care.

**Abstract:**

**Background**: Parents of children undergoing liver transplantation face substantial caregiving demands that may adversely affect their mental health across multiple domains. Systematic evaluation of psychosocial outcomes in this population remains limited, particularly in settings that include immigrant families. **Method**: This was a single-center, cross-sectional study including the parents of 50 children after liver transplantation. Major sociodemographic variables included parental age, sex, education, chronic disease, and immigration status. We also recorded children’s demographics, transplant-related data, follow-up findings, and mental health status. Instruments for psychiatric assessment included the Generalized Anxiety Disorder 7-item scale (GAD-7; anxiety), Patient Health Questionnaire-9 (PHQ-9; depression), Perceived Stress Scale-10 (PSS-10; stress), and Pittsburgh Sleep Quality Index (PSQI; sleep quality). **Results**: We enrolled 50 parents of 50 pediatric liver transplant recipients (43 Turkish citizens, 7 Syrian immigrants; 28 fathers, 22 mothers; mean age: 40.10 ± 6.65). Time since transplantation showed weak negative correlation with PHQ-9 and GAD-7. Stress (PSS) levels had weak to strong positive correlation with PSQI, PHQ-9, and GAD-7. Sleep quality (PSQI) was positively correlated with PHQ-9 and GAD-7. Depressive findings (PHQ-9) were strongly and positively correlated with GAD-7. In Firth-penalized multivariable models, high PHQ-9 scores were independently associated with shorter time since transplantation (*p* = 0.001) and high PSS (*p* = 0.003). High GAD-7 scores were independently associated with shorter time since transplantation (*p* = 0.025) and high PSS (*p* = 0.001). **Conclusions**: The parents of pediatric liver transplant recipients experience high levels of stress, sleep issues, depression, and anxiety, which demonstrate multiple correlations.

## 1. Introduction

Liver transplantation is a life-saving procedure for end-stage liver disease, including biliary atresia, metabolic disorders, and acute liver failure [1]. Advances in surgical techniques, immunosuppression, and perioperative care have greatly increased survival rates in both adults and children [2,3]. Improved survival has redirected clinical emphasis from solely transplant success to other outcomes such as psychiatric, developmental, and psychosocial well-being [4,5]. Understanding the specific mental health consequences for parents across different phases of the transplant process has therefore become an emerging clinical priority.

Despite these improvements, the transplant process places significant psychological stress on families, especially parents, who must manage uncertainties regarding their child’s prognosis, potential complications, medication adherence, frequent follow-ups, infection risks, and demanding caregiving responsibilities [6,7]. The experience of liver transplantation is frequently described as a traumatic life event that significantly affects parental mental health, resulting in increased anxiety, depression, post-traumatic stress disorder, sleep disturbances, and perceived stress [8,9]. Research demonstrates that acute post-transplant phases, characterized by intensive care admissions, life-threatening issues, and fears of organ rejection, increase parental distress [2,5]. Parental mental health is also crucial to ensure better transplant outcomes since elevated parental anxiety and depression are associated with poor mental health, treatment non-adherence, and complications [1,10]. As such, it is evident that systematic evaluations of parental psychosocial status can improve clinical care. However, current research primarily utilizes scale-based assessments with restricted samples and frequently omits analysis of social vulnerabilities, including immigration status [3,11].

Turkey is home to more than 3.5 million Syrians, many of whom are children [12,13]. Immigration status exacerbates psychiatric risks for parents due to war-related trauma, socioeconomic difficulties, housing instability, financial stress, language barriers, healthcare access issues, and insufficient social support [7,14]. Immigration also complicates health service utilization and disrupts family dynamics, which adversely impact management of chronic illnesses [15,16]. Evidence is limited regarding the relationships between immigration and pediatric liver transplantation [17]. To our knowledge, no prior study has simultaneously examined parental mental health outcomes—encompassing anxiety, depression, perceived stress, and sleep quality—across the post-transplant period while explicitly accounting for immigration status. Given the global rise in refugee populations and Turkey’s position as the world’s largest host country for Syrian refugees, this constitutes a clinically meaningful gap in the literature [18,19].

Addressing this gap, the present study represents one of the first assessments of psychosocial burden among parents of pediatric liver transplant recipients conducted in a clinical setting with a substantial immigrant patient population. With this cross-sectional study we sought to assess anxiety, depression, perceived stress, and sleep quality among the parents of children who underwent pediatric liver transplantation. Our secondary aim was to compare the experiences of Syrian immigrant parents to those of Turkish citizens.

## 2. Materials and Methods

### 2.1. Population and Setting

The study population consisted of parents of children who had received pediatric liver transplantation at our center. The inclusion criteria were established as follows: parents aged 18 years or older whose child had undergone a liver transplant at any phase of the process (including acute post-transplant periods); proficiency in Turkish or Arabic for effective communication; voluntary participation; and willingness to undergo evaluation irrespective of the duration since transplantation. Exclusion criteria encompassed: severe psychotic symptoms that hindered the capacity to complete assessments; substantial cognitive impairment obstructing informed consent or questionnaire completion; active suicidal ideation necessitating immediate intervention; and insurmountable language or cultural barriers that could not be mitigated through Arabic translation assistance offered by trained research personnel. Due to the scarcity of the pediatric liver transplant population and the emphasis on a vulnerable subgroup (including immigrant families), the composition of the immigrant subgroup (*n* = 7) reflects the actual proportion of Syrian families attending our transplant center during the study period and was not predetermined by design. Given this limitation, comparisons involving immigration status are presented as exploratory findings only.

### 2.2. Study Design and Ethics

This study utilized a single-center, cross-sectional, descriptive design, conducted at the Liver Transplantation Institute, İnönü University Faculty of Medicine, Malatya, Turkey. Data collection continued from January 2025 to January 2026, during standard outpatient follow-up appointments or inpatient admissions at the transplant center. Ethical approval was obtained from the local ethics committee (Date: 2 December 2025, No: 2025/8674) based on the ethical standards described in the Declaration of Helsinki. Before enrollment, all parents who took part in the study had to sign written informed consent forms. The study’s purposes, procedures, potential risks, and benefits were explained to all participants and they were informed of their right to withdraw at any time.

### 2.3. Demographics and Clinical Data

The data collection was carefully planned in two stages to obtain self-reported psychological measures and important clinical and sociodemographic variables. All procedures were conducted by trained research personnel in person at the transplant center, with Arabic translation assistance provided for Syrian immigrant parents to alleviate language barriers and improve accuracy. When needed, questionnaires were given verbally to illiterate individuals, and forms were checked right away to ensure completeness. Parental reports and electronic medical records were used to double-check clinical information.

A researcher-created clinical information form was used to record sociodemographic and clinical data. This included the age and sex of the parent, their level of education (literate, primary school, secondary school, high school, associate degree, or bachelor’s degree), whether they had a chronic disease, the child’s age and sex, any comorbidities in the child, the age at transplantation, the time since transplantation, type of donor (living or deceased), the immunosuppressive therapy (e.g., tacrolimus or everolimus), transplantation-related complications, rejection events, hospitalizations, psychiatric follow-up and diagnoses of the child, and immigration status (Turkish citizen or Syrian immigrant). It was also noted that economic status and access to health services could be stressors. These variables were documented via a combination of parental self-report during interviews and the examination of hospital records, ensuring consistency and comprehensiveness.

All parents denied any prior psychiatric diagnosis, as confirmed through direct clinical interview and medical record review. Pre-existing psychiatric morbidity was therefore not a confounding factor in the present sample. Detailed socioeconomic data were not collected; however, the study population was relatively homogeneous in this regard, with all participants belonging to lower-middle socioeconomic strata based on clinical observation and the demographic profile of the transplant center’s catchment area. This relative homogeneity may have reduced, though not eliminated, socioeconomic confounding.

### 2.4. Evaluation of Psychological Outcomes

The Generalized Anxiety Disorder 7-item scale (GAD-7) was used to measure parental anxiety. This is a validated 7-item self-report tool that grades anxiety symptoms experienced in the past two weeks. The total score ranges from 0 to 21 points, with each item scored on a 4-point Likert scale (0 = not at all, 3 = nearly every day). The following cut-off points were used: 0–4 (minimal anxiety), 5–9 (mild), 10–14 (moderate), and 15–21 (severe) [20].

The Patient Health Questionnaire-9 (PHQ-9) was used to measure depression. It is a 9-item scale that follows DSM criteria for major depressive disorder and evaluates symptoms from the past two weeks. Each item is on a Likert scale from 0 to 3 points. Total scores range from 0 to 27 points, with classification into five groups: 0 to 4 (minimal depression), 5 to 9 (mild), 10 to 14 (moderate), 15 to 19 (moderately severe), and 20 to 27 (severe) [21].

The 10-item Perceived Stress Scale (PSS-10) was used to measure stress. This scale questions individuals to assess how stressful they think their life has been in the past month. Responses for each item are scored from 0 (never) to 4 (very often), and total scores range from 0 to 40 points. A score of 0–13 indicates low stress, 14–26 indicates moderate stress, and 27–40 indicates high stress [22].

The Pittsburgh Sleep Quality Index (PSQI) is a 19-item questionnaire that asks about seven aspects of sleep over the past month (subjective sleep quality, latency, duration, efficiency, disturbances, medication use, and daytime dysfunction). Scores on a global scale go from 0 to 21, and scores exceeding 5 indicate poor sleep quality [23].

### 2.5. Statistics

IBM SPSS Statistics for Windows, Version 27 was used for most analyses (IBM Corp., Armonk, NY, USA); Firth-penalized logistic regression was performed in R using the logistf package to address complete separation in the regression model. The accepted value for statistical significance was *p* < 0.05. The Shapiro–Wilk test was used to check normality. Descriptives for numerical variables were provided as mean ± standard deviation (parametric) or median (25th–75th percentile) (non-parametric). Categorical variables were described with absolute and relative frequency. Student's *t*-test or Mann–Whitney U test was used to compare continuous variables between groups, depending on the assumption of normality. We used Fisher’s exact or the Fisher–Freeman–Halton tests to analyze categorical variable distributions. Given the substantial imbalance between subgroups (n = 43 Turkish citizens vs. n = 7 Syrian immigrants), formal between-group statistical comparisons by immigration status were not conducted: descriptive characteristics by immigrant status are presented in Table 1 and Table 2 for transparency only and are not interpreted inferentially. To assess the relationships between continuous variables, Pearson or Spearman correlation coefficients were computed. Univariable and multivariable logistic regression analyses were conducted to identify factors independently associated with PHQ-9 and GAD-7 scores. To address concerns regarding small-sample bias and model stability in the multivariable analyses, where the number of outcome events was modest relative to the number of candidate predictors, the multivariable models were estimated using Firth’s penalized maximum likelihood method, which is specifically recommended for logistic regression with small samples or sparse events as it provides reduced-bias estimates and yields finite parameter estimates even in the presence of separation. The final multivariable models retained the candidate predictors that reached significance at the univariable level. Given the cross-sectional design, all findings are interpreted as associations, and no causal inferences are drawn. PHQ-9 and GAD-7 scores were dichotomized at ≥5 to identify parents with at least mild symptomatic burden, in accordance with the severity categories established in the original validation studies of these instruments [20,21]. This threshold was selected to maximize sensitivity for detecting early psychosocial distress in a clinical caregiving population, where subthreshold symptoms may carry clinical significance even in the absence of a formal diagnosis.

Given the exploratory nature of this study and the scarcity of the target population at a single transplant center, an a priori power calculation was not conducted. A post hoc sensitivity analysis indicated that with n = 50 and α = 0.05, the study had adequate power (>80%) to detect medium-to-large correlations (r ≥ 0.35) but was underpowered to detect small-to-medium between-group differences, particularly given the imbalanced immigrant subgroup.

## 3. Results

Overall, the study population demonstrated a substantial psychosocial burden. The majority of parents (66%) reported moderate perceived stress levels, and 46% had poor sleep quality (PSQI > 5). Depressive symptoms of at least mild severity (PHQ-9 ≥ 5) were present in 36% of parents, and at least mild anxiety (GAD-7 ≥ 5) was identified in 30%. Descriptive characteristics by immigrant status are summarized in Table 1 and Table 2; as outlined in the Methods, formal between-group statistical comparisons by immigration status were not conducted given the substantial subgroup imbalance. The following sections present correlation and multivariable findings in detail.

There were 50 parents in the study. Forty-three (86%) were Turkish citizens and 7 (14%) were Syrian immigrants. The mean age of the parents was 40.10 ± 6.65 years (Turkish citizens: 40.47 ± 6.37 years; Syrian immigrants: 37.86 ± 8.36 years). There were 22 (44%) mothers and 28 (56%) fathers in total. The median age of the children was 14 years (11–16 years). Sociodemographic and clinical characteristics of parents and children, stratified by immigrant status, are summarized descriptively in Table 1. Psychosocial assessment scores and the corresponding symptom severity distributions, also stratified by immigrant status, are presented in Table 2. As outlined in the Methods, formal between-group statistical comparisons by immigration status were not conducted given the substantial subgroup imbalance, and Table 1 and Table 2 should be interpreted as descriptive summaries only.

Parental education level was positively correlated with PSQI score (r = 0.349, *p* = 0.013). Time elapsed since transplantation was negatively correlated with both the PHQ-9 score (r = −0.357, *p* = 0.011) and the GAD-7 score (r = −0.328, *p* = 0.020). The PSS score had a positive correlation with the PSQI score (r = 0.346, *p* = 0.014), the PHQ-9 score (r = 0.521, *p* < 0.001), and the GAD-7 score (r = 0.636, *p* < 0.001). The PSQI score exhibited a positive correlation with the PHQ-9 score (r = 0.343, *p* = 0.015) and the GAD-7 score (r = 0.331, *p* = 0.019). The PHQ-9 scores were strongly and positively correlated with the GAD-7 score (r = 0.787, *p* < 0.001) (Table 3).

Firth-penalized multivariable logistic regression analysis showed that a shorter time interval since transplantation (OR: 0.726, 95% CI: 0.539–0.889, *p* = 0.001) and a high PSS score (OR: 1.317, 95% CI: 1.080–1.738, *p* = 0.003) were independently associated with a high (≥5) PHQ-9 score (Table 4).

Similarly, in the Firth-penalized model, a shorter time interval since transplantation (OR: 0.802, 95% CI: 0.618–0.975, *p* = 0.025) and an elevated PSS score (OR: 1.452, 95% CI: 1.150–2.018, *p* = 0.006) were independently associated with a high (≥5) GAD-7 score (Table 5).

## 4. Discussion

Overall, the study population demonstrated a substantial psychosocial burden. The majority of parents (66%) reported moderate perceived stress levels, and 46% had poor sleep quality (PSQI > 5). Depressive symptoms of at least mild severity (PHQ-9 ≥ 5) were present in 36% of parents, and at least mild anxiety (GAD-7 ≥ 5) was identified in 30%. The following sections present correlation and multivariable findings in detail. The main results showed that there were positive links between parental education and sleep quality problems, as well as strong links between stress, sleep, depression, and anxiety measures. Moreover, we found that longer time elapsed since transplantation was independently associated, in Firth-penalized models, with lower levels of depression and anxiety, whereas higher perceived stress was independently associated with greater depression and anxiety scores.

Immigrant families often face greater levels of psychosocial stress that arise from migration-related traumas and systemic obstacles, as demonstrated by previous research on refugee populations [18]. Existing research indicates increased mental health risks among immigrant caregivers; however, our findings offer a more complex perspective within the context of pediatric liver transplantation. We found similar psychological outcomes in immigrant and non-immigrant parents, perhaps in relation with the greater severity and complexity of transplant experiences relative to other pediatric chronic illnesses. Immigrant parents, especially Syrian refugees in Turkey, frequently face war-related trauma, socioeconomic instability, language barriers, and limited access to healthcare [18,24]. Eruyar et al. [7] found that Syrian refugee children in Turkey exhibited significant mental health issues influenced by parental factors, with relational dynamics being a crucial determinant of child outcomes. Yayan et al. [11] noted increased post-traumatic stress, depression, and anxiety in Syrian refugee children, linking these conditions to familial stressors that indirectly impact parental well-being. In our cohort, the descriptive similarity of psychosocial scores across Turkish and Syrian immigrant parents is consistent with the possibility that the transplant process itself represents an overriding source of burden for all parents; however, this interpretation is purely speculative as no formal between-group statistical comparisons were performed and the immigrant subgroup was very small. It is also possible that the availability of translation services in hospitals and the amount of time dedicated to transplantation patients may remedy the adverse impacts of immigration [16]. Given the critically small size of the immigrant subgroup (n = 7), all comparisons between groups must be considered exploratory and hypothesis-generating rather than conclusive. In particular, the descriptive similarity of values across subgroups should not be interpreted as evidence of equivalence (no formal between-group statistical comparisons were performed), as the study was substantially underpowered for between-group comparisons, and a Type II error cannot be excluded. These results should not be interpreted as evidence of the absence of immigration-related psychosocial differences, and replication in adequately powered, multicenter cohorts is essential. This finding, derived from a critically small and unbalanced subgroup, does not support generalizable conclusions about the relationship between immigration status and psychosocial burden. It is presented solely as a hypothesis-generating observation to motivate future research in adequately powered, multicenter cohorts [2].

Studies on caregivers of children with chronic conditions, including organ transplants, consistently demonstrate bidirectional relationships between perceived stress and disturbances in sleep and emotional well-being [25]. These related domains impact individual well-being, family functioning, and child outcomes, thereby requiring a comprehensive analysis of their interactions [4]. The positive correlations between perceived stress, sleep quality, depression, and anxiety are evidence for the overarching mental burden experienced by parents, which is also consistent with evidence indicating that chronic caregiving adversely affects various health domains. These relationships reflect the findings of Andersen et al. [5], who identified correlations between child sleep disturbances and diminished parental health-related quality of life in liver transplant families, with sleep issues identified to be a mediator for emotional distress. In a similar study, Savsar et al. [25] found that 65% of adults who had liver transplants had trouble sleeping, which was linked to anxiety about the future. Our results indicate that caregivers suffer from similar problems. Forner-Puntonet et al. [4] conducted a study indicating that families of pediatric solid organ transplant recipients experienced elevated anxiety and avoidance coping, with elevated stress worsening outcomes. In our mixed cohort of immigrant and non-immigrant parents we observed that high PSS was independently associated with increased PHQ-9 and GAD-7 scores. These associations were estimated using Firth’s penalized maximum likelihood method, which mitigates small-sample bias in logistic regression; nevertheless, given the modest sample size, external validation in larger, adequately powered cohorts remains essential before these associations can be considered firmly established. These findings suggest that interventions addressing perceived stress may also be relevant to sleep and emotional well-being in this population. It should be noted that all psychological constructs in this study were measured using self-report instruments administered in the same session. The correlations observed between perceived stress, sleep quality, depression, and anxiety—including the particularly strong association between PHQ-9 and GAD-7 scores (r = 0.787)—may be partially inflated by shared method variance arising from common source bias. The true magnitude of these associations may therefore differ from the estimates reported here, and the results should be interpreted accordingly.

The temporal aspect of recovery in chronic pediatric conditions frequently indicates gradual enhancements in caregiver mental health as medical stability improves; however, socioeconomic factors, such as education, can create variability in adaptation processes [3,9]. The inverse correlation between time elapsed since transplantation and depression or anxiety scores is consistent with a pattern of gradual psychosocial adjustment, although causal inference is precluded by the cross-sectional design. Interestingly, Duvant et al. [9] reported that transplanted children and parents had better quality of life than those with other chronic conditions, which is broadly consistent with the pattern of decreasing psychosocial distress over time observed in our data. In partial support, Parmar et al. [3] found that older age at transplantation was linked to worse health-related quality of life, while Miserachs et al. [2] reported that improved coping and adaptation were fundamental to better outcomes. Parental education exhibited a positive correlation with the PSQI, indicating that caregivers with higher education levels may encounter increased sleep disturbances due to heightened risk awareness, which is in line with prior evidence regarding the effects of cognitive impairment among pediatric transplant recipients [26].

The present study did not evaluate social support, coping strategies, or broader family functioning, all of which have been identified as important moderators of caregiver distress in transplant populations. Forner-Puntonet et al. [4] highlighted the role of coping strategies—particularly avoidance—in shaping psychosocial outcomes among families of pediatric solid organ transplant recipients, while Fredericks et al. [10] identified family functioning as a key predictor of adherence and psychological outcomes. The incorporation of these constructs in future studies would enable more comprehensive modeling of the pathways through which transplant-related stressors translate into parental distress, and would facilitate the design of targeted family-centered interventions.

There are a number of important limitations. The small sample size, especially the immigrant subgroup (n = 7), significantly diminishes statistical power, increasing the possibility of Type II errors and potentially obscuring differences in psychosocial outcomes between groups. The substantial numerical imbalance between the two groups (n = 43 vs. n = 7) further increases the risk of Type II error in between-group comparisons, and the descriptive similarity of values across subgroups should not be interpreted as evidence of equivalence (no formal between-group statistical comparisons were performed) [13]. The number of outcome events was modest relative to the number of candidate predictors in the multivariable models, which can compromise the stability of conventional maximum-likelihood estimates. To mitigate this concern, the multivariable models were estimated using Firth’s penalized maximum likelihood method, which provides reduced-bias estimates and is specifically recommended for logistic regression with small samples or sparse events. Even so, the modest sample size remains a fundamental limitation, and the reported odds ratios and confidence intervals should be regarded as preliminary estimates requiring external validation in larger, independent cohorts. Furthermore, the cross-sectional design precludes causal interpretation of all observed associations, including the relationship between perceived stress and depression or anxiety outcomes. Accordingly, all language in the Results, Discussion, and Conclusions of this paper reflects associations only, and no directional or causal claims are made or implied. Reliance on self-report instruments introduces the possibility of recall and social desirability bias, which may be particularly pronounced in populations carrying mental health stigma. No data were collected on pre-transplant parental psychiatric history, objective socioeconomic status, or level of social support—all of which represent potential confounders that could have influenced the results. Child-reported outcomes were not obtained, preventing assessment of whether parental perceptions align with child experience. There is a need for extensive, longitudinal, multicenter studies employing objective metrics and a variety of instruments to effectively examine both parental and child experiences before, during and after liver transplantation.

## 5. Conclusions

This study illustrates that parents of children who underwent pediatric liver transplantation experience psychosocial challenges, including stress, sleep disturbances, depression, and anxiety. Descriptive characteristics by immigrant status are reported in this study; however, given the substantial imbalance between subgroup sizes, formal between-group comparisons by immigration status were not performed, and the present data should not be interpreted as evidence either of equivalence or of difference between Turkish and Syrian immigrant parents. Crucially, in Firth-penalized multivariable models, shorter time since transplantation and higher perceived stress were each independently associated with elevated depression and anxiety scores. The strong positive associations among stress, sleep disturbance, depression, and anxiety are consistent with a closely interconnected psychosocial burden profile, though the directionality and causal structure of these relationships cannot be determined from cross-sectional data. We believe there is a need to improve mental health interventions for the parents of children scheduled for liver transplantation to ensure that the psychiatric burdens do not progress into overt disease. Whether such interventions would improve adaptation and long-term outcomes in children as well as parents remains to be established in prospective studies.

## Figures and Tables

**Table 1:** Sociodemographic and clinical characteristics of parents and children by immigrant status.

	Total (n = 50)	Immigrant	
		No (n = 43)	Yes (n = 7)
Parent			
Mother	22 (44.00%)	18 (41.86%)	4 (57.14%)
Father	28 (56.00%)	25 (58.14%)	3 (42.86%)
Age of parent, years	40.10 ± 6.65	40.47 ± 6.37	37.86 ± 8.36
Education status of parent			
Literate	10 (20.00%)	7 (16.28%)	3 (42.86%)
Primary school	17 (34.00%)	16 (37.21%)	1 (14.29%)
Secondary school	12 (24.00%)	10 (23.26%)	2 (28.57%)
High school	6 (12.00%)	5 (11.63%)	1 (14.29%)
Associate degree	2 (4.00%)	2 (4.65%)	0 (0.00%)
Bachelor degree	3 (6.00%)	3 (6.98%)	0 (0.00%)
Chronic disease in parent	10 (20.00%)	9 (20.93%)	1 (14.29%)
Age of child, years	14 (11–16)	14 (11–16)	14 (8–16)
Sex of child			
Male	28 (56.00%)	25 (58.14%)	3 (42.86%)
Female	22 (44.00%)	18 (41.86%)	4 (57.14%)
Comorbidity in child	2 (4.00%)	2 (4.65%)	0 (0.00%)
Age at transplantation, years	5 (2–11)	4 (2–10)	7 (3–13)
Time since transplantation, years	7.44 (2.25–11.67)	9.07 (3.18–11.85)	4.58 (0.50–6.91)
Type of donor			
Living	44 (88.00%)	37 (86.05%)	7 (100.00%)
Deceased	6 (12.00%)	6 (13.95%)	0 (0.00%)
Immunosuppressive drug			
Tacrolimus	43 (86.00%)	37 (86.05%)	6 (85.71%)
Everolimus	7 (14.00%)	6 (13.95%)	1 (14.29%)
Transplantation complication	0 (0.00%)	0 (0.00%)	0 (0.00%)
Rejection attack, last year	0 (0.00%)	0 (0.00%)	0 (0.00%)
Hospitalization, last year	2 (4.00%)	2 (4.65%)	0 (0.00%)
Psychiatric follow-up for child	1 (2.00%)	1 (2.33%)	0 (0.00%)
Psychiatric diagnosis in child	0 (0.00%)	0 (0.00%)	0 (0.00%)

Descriptive statistics are presented using mean ± standard deviation for normally distributed continuous variables, median (25th percentile–75th percentile) for non-normally distributed continuous variables and frequency (percentage) for categorical variables. *p*-values reflect exploratory between-group comparisons (Turkish citizens vs. Syrian immigrants) and are presented for descriptive completeness only. Given the extreme imbalance between groups (n = 43 vs. n = 7), these comparisons are substantially underpowered and should not be interpreted as inferential statistical tests.

**Table 2:** Psychosocial assessment scores and symptom severity distributions by immigrant status.

	Total (n = 50)	Immigrant	
		No (n = 43)	Yes (n = 7)
PSS score	15.20 ± 5.64	14.79 ± 5.55	17.71 ± 5.94
Low stress (0–13)	16 (32.00%)	15 (34.88%)	1 (14.29%)
Moderate stress (14–26)	33 (66.00%)	28 (65.12%)	5 (71.43%)
High stress (27–40)	1 (2.00%)	0 (0.00%)	1 (14.29%)
PSQI score	5 (3–8)	5 (3–8)	6 (2–7)
Normal (0–5)	27 (54.00%)	25 (58.14%)	2 (28.57%)
High (>5)	23 (46.00%)	18 (41.86%)	5 (71.43%)
PHQ-9 score	1 (0–8)	1 (0–8)	0 (0–12)
Minimal (0–4)	32 (64.00%)	28 (65.12%)	4 (57.14%)
Mild (5–9)	12 (24.00%)	11 (25.58%)	1 (14.29%)
Moderate (10–14)	6 (12.00%)	4 (9.30%)	2 (28.57%)
Moderately severe (15–19)	0 (0.00%)	0 (0.00%)	0 (0.00%)
Severe (20–27)	0 (0.00%)	0 (0.00%)	0 (0.00%)
GAD-7 score	0 (0–5)	0 (0–5)	0 (0–12)
Minimal (0–4)	35 (70.00%)	30 (69.77%)	5 (71.43%)
Mild (5–9)	9 (18.00%)	9 (20.93%)	0 (0.00%)
Moderate (10–14)	4 (8.00%)	2 (4.65%)	2 (28.57%)
Severe (15–21)	2 (4.00%)	2 (4.65%)	0 (0.00%)

Descriptive statistics are presented using mean ± standard deviation for normally distributed continuous variables, median (25th percentile–75th percentile) for non-normally distributed continuous variables and frequency (percentage) for categorical variables. Abbreviations: GAD-7: Generalized Anxiety Disorder 7-item; PSS: Perceived Stress Scale; PSQI: Pittsburgh Sleep Quality Index; PHQ-9: Patient Health Questionnaire 9-item.

**Table 3:** Correlations between demographics and assessment scores.

		PSS Score	PSQI Score	PHQ-9 Score	GAD-7 Score
Age of parent, years	r	0.037 ^†^	0.038 ^‡^	−0.164 ^‡^	0.020 ^‡^
	*p*	0.798	0.795	0.254	0.888
Education status of parent	r	0.117 ^‡^	0.349 ^‡^	0.221 ^‡^	0.268 ^‡^
	*p*	0.418	**0.013**	0.123	0.060
Age of child, years	r	−0.105 ^‡^	−0.090 ^‡^	−0.271 ^‡^	−0.171 ^‡^
	*p*	0.466	0.534	0.057	0.235
Age at transplantation, years	r	0.082 ^‡^	−0.040 ^‡^	0.160 ^‡^	0.197 ^‡^
	*p*	0.571	0.781	0.266	0.171
Time since transplantation, years	r	−0.168 ^‡^	0.036 ^‡^	−0.357 ^‡^	−0.328 ^‡^
	*p*	0.244	0.805	**0.011**	**0.020**
PSS score	r		0.346 ^‡^	0.521 ^‡^	0.636 ^‡^
	*p*		**0.014**	**<0.001**	**<0.001**
PSQI score	r			0.343 ^‡^	0.331 ^‡^
	*p*			**0.015**	**0.019**
PHQ-9 score	r				0.787 ^‡^
	*p*				**<0.001**

^†^ Pearson correlation coefficient, ^‡^ Spearman correlation coefficient. Statistically significant *p* values are shown in bold. Abbreviations: GAD-7: Generalized Anxiety Disorder 7-item; PSS: Perceived Stress Scale; PSQI: Pittsburgh Sleep Quality Index; PHQ-9: Patient Health Questionnaire 9-item; r: Correlation coefficient.

**Table 4:** Odds ratios for high (≥5) PHQ-9 score, logistic regression analysis results.

	Univariable		Multivariable	
	OR (95% CI)	*p*	OR (95% CI)	*p*
Immigrant, Yes	1.400 (0.276–7.096)	0.685		
Parent, Mother	1.462 (0.457–4.674)	0.522		
Age of parent, years	0.996 (0.913–1.088)	0.936		
Education status of parent	1.585 (1.002–2.507)	**0.049**	1.368 (0.761–2.561)	0.295
Chronic disease in parent, Yes	2.077 (0.510–8.466)	0.308		
Age of child, years	0.829 (0.680–1.011)	0.065		
Sex of child, Male	0.972 (0.304–3.110)	0.962		
Comorbidity in child, Yes	1.824 (0.107–31.031)	0.678		
Age at transplantation, years	1.107 (0.980–1.251)	0.101		
Time since transplantation, years	0.812 (0.704–0.937)	**0.004**	0.726 (0.539–0.889)	**0.001**
Type of donor, Deceased	1.933 (0.347–10.769)	0.452		
Immunosuppressive drug, Everolimus	2.762 (0.543–14.057)	0.221		
Hospitalization, last year	0.000 (0.000–N/A)	0.999		
Psychiatric follow-up for child, Yes	0.000 (0.000–N/A)	1.000		
PSS score	1.343 (1.112–1.622)	**0.002**	1.317 (1.080–1.738)	**0.003**
PSQI score	1.283 (1.053–1.564)	**0.014**	1.249 (0.952–1.740)	0.110

Statistically significant *p* values are shown in bold. Multivariable estimates are derived from Firth’s penalized maximum likelihood logistic regression. Abbreviations: CI: Confidence interval; N/A: Non-applicable; OR: Odds ratio; PSS: Perceived Stress Scale; PSQI: Pittsburgh Sleep Quality Index; PHQ-9: Patient Health Questionnaire 9-item.

**Table 5:** Odds ratios for high (≥5) GAD-7 score, logistic regression analysis results.

	Univariable		Multivariable	
	OR (95% CI)	*p*	OR (95% CI)	*p*
Immigrant, Yes	0.923 (0.158–5.388)	0.929		
Parent, Mother	1.714 (0.507–5.802)	0.386		
Age of parent, years	1.001 (0.913–1.097)	0.981		
Education status of parent	1.457 (0.927–2.290)	0.103		
Chronic disease in parent, Yes	1.758 (0.415–7.441)	0.444		
Age of child, years	0.910 (0.746–1.109)	0.349		
Sex of child, Male	0.583 (0.172–1.974)	0.386		
Comorbidity in child, Yes	2.429 (0.142–41.601)	0.540		
Age at transplantation, years	1.087 (0.960–1.231)	0.188		
Time since transplantation, years	0.874 (0.763–1.000)	**0.049**	0.802 (0.646–0.997)	**0.025**
Type of donor, Deceased	2.667 (0.472–15.078)	0.267		
Immunosuppressive drug, Everolimus	1.937 (0.376–9.974)	0.429		
Hospitalization, last year	0.000 (0.000–N/A)	0.999		
Psychiatric follow-up for child, Yes	0.000 (0.000–N/A)	1.000		
PSS score	1.543 (1.191–1.998)	**0.001**	1.452 (1.114–1.892)	**0.001**
PSQI score	1.336 (1.082–1.651)	**0.007**	1.257 (0.965–1.637)	0.063

Statistically significant *p* values are shown in bold. Multivariable estimates are derived from Firth’s penalized maximum likelihood logistic regression. Abbreviations: CI: Confidence interval; N/A: Non-applicable; OR: Odds ratio; PSS: Perceived Stress Scale; PSQI: Pittsburgh Sleep Quality Index; PHQ-9: Patient Health Questionnaire 9-item.

## Data Availability

The data presented in this study are available from the corresponding author upon reasonable request due to privacy and ethical restrictions.

## Abbreviations

LT	Liver Transplantation
GAD-7	Generalized Anxiety Disorder 7-item scale
PHQ-9	Patient Health Questionnaire-9
PSS-10	Perceived Stress Scale-10
PSQI	Pittsburgh Sleep Quality Index
SPSS	Statistical Package for the Social Sciences
